# Gender differences in oral health among prisoners: a cross-sectional study from Taiwan

**DOI:** 10.1186/s12903-023-03598-4

**Published:** 2023-11-21

**Authors:** Yu-Pei Yang, Ho-Tsung Hsin, Bing-Long Wang, Yen-Chun Wang, Pi-Ching Yu, Shi‑Hao Huang, Ren‑Jei Chung, Yao-Ching Huang, Tao-Hsin Tung

**Affiliations:** 1 Department of Hematology, Taizhou Hospital of Zhejiang Province Affiliated to Wenzhou Medical University, Linhai, Zhejiang 317000 China; 2 Evidence-Based Medicine Center, Taizhou Hospital of Zhejiang Province Affiliated to Wenzhou Medical University, Linhai, Zhejiang 317000 China; 3 Department of Critical Care Medicine, Cardiovascular Intensive Care Unit, Far-Eastern Memorial Hospital, New Taipei City, 10602 Taiwan; 4 Department of Mechanical Engineering, Yuan Ze University, Taoyoung, 32003 Taiwan; 5 School of Health Policy and Management, Chinese Academy of Medical Sciences & Peking Union Medical College, Beijing, 100730 China; 6 Department of Public Health, College of Health Science, Kaohsiung Medical University, Kaohsiung, 80708 Taiwan; 7 School of Public Health, National Defense Medical Center, Taipei, 11490 Taiwan; 8 Department of Chemical Engineering and Biotechnology, National Taipei University of Technology (Taipei Tech), Taipei, 10608 Taiwan; 9 School of Management, Putian University, Putian, 351200 China

**Keywords:** Taiwan prison, Oral Disease, Sex difference, Age difference

## Abstract

**Background:**

The prevalence of oral diseases among Taiwanese prisoners has rarely been investigated. This study aimed to estimate the gender-specific prevalence of oral disease in a sample of Taiwanese prisoners.

**Methods:**

We included 83,048 participants from the National Health Insurance (NHI) Program. Outcomes were measured using the clinical version of the International Classification of Diseases, Ninth Revision (ICD-9-CM). For prevalence, we provide absolute values and percentages. We also performed a χ2 test to assess sex and age group differences in the percentage of disease in the oral cavity, salivary glands, and jaw.

**Results:**

The prevalence rate of oral diseases was 25.90%, which was higher than that of the general population. The prevalence of oral diseases in female prisoners was higher than that in male prisoners (*p* < 0.001), and the prevalence of oral diseases in prisoners aged ≤ 40 was higher than that of prisoners aged > 40. Among all cases of diagnosed oral diseases, the top three diseases were dental hard tissue diseases (13.28%), other cellulitis and abscesses (9.79%), and pruritus and related conditions (2.88%), respectively. The prevalence of various oral diseases in female prisoners was significantly higher than that in male prisoners.

**Conclusion:**

Oral disease is common among Taiwanese prisoners. Female prisoners had a higher prevalence of oral, salivary gland, and jaw diseases than male prisoners. Therefore, early prevention and appropriate treatment are required and also a need for gender-specific oral disease products given the differences in the prevalence of oral disease among male and female prisoners.

**Supplementary Information:**

The online version contains supplementary material available at 10.1186/s12903-023-03598-4.

## Key points

1. The prevalence of oral diseases in Taiwanese prisoners is higher than that of the general population.
2. Dental hard tissue diseases and gingival and periodontal diseases are the most prevalent oral diseases among male and female prisoners.
3. The results indicate the need for early prevention and appropriate treatment of oral diseases in Taiwan's criminal justice system.

**Supplementary Information:**

The online version contains supplementary material available at 10.1186/s12903-023-03598-4.

## Introduction

World Health Organization (WHO) estimates that nearly 300 million people worldwide (about 5% of the population) suffer from one form or another of oral disease [1]. The global burden of oral diseases exceeds the global burden of the next five most prevalent noncommunicable diseases combined by nearly 100 million cases [2]. Common oral diseases in Taiwan include dental caries, periodontal disease, and oral cancer [3]. The National Oral Health Plan aims to reduce the prevalence of dental caries, caries index, prevalence of periodontal disease, oral cancer, and other oral mucosal lesions [4]. However, according to relevant statistical reports and research reports in recent years, the oral health indicators of some Chinese people are not ideal, and the use of preventive services is not good [5]. The breakdown is as follows: The dental caries rate of 1.5-year-old children exceeds the target set by WHO [6]. The prevalence rate of the disease is relatively high, and the number of oral cancer-related cancer cases is increasing year by year [7].

A prison is a place where prisoners are detained, and prisoners are subject to restrictions and deprivations of various liberties under state power as punishment for various crimes [8]. Prisons are most commonly used in the criminal justice system, where people accused of a crime may be incarcerated until trial [9]. Those who plead guilty at trial or are found guilty may be sentenced to a prison sentence [10]. According to the latest statistics from the Ministry of Justice, as of July 2023, there were more than 82,949 people housed in correctional institutions, while Taiwan’s legal housing capacity is only 60,375 people [11].

The majority of inmates currently in prison commit drug crimes, followed by property crimes–theft [12]. The overcrowded prison population exploded, resulting in the deterioration of the prison environment [13]. The first problem caused by the overcrowding phenomenon is the rapidly deteriorating quality of life in prisons: crowding, stuffy heat, diluted medical resources, and poor public health environment, cause adverse reactions such as unstable prison conditions; correspondingly, high-pressure and intensive guarding must be used instead [14]. To avoid possible disorder and riots, in this case, the teachings, homework, and legal rights (such as correspondence, and complaints) that are the basis for helping prisoners return to society are transformed into a correctional orientation, and even deprivation and prohibition become a part of high-pressure management [15].

Many prisoners enter prison in poor oral health and require emergency treatment, possibly due to a lack of knowledge of good oral health practices. Substance abuse can lead to severe tooth decay and gum disease [16]. Tobacco use increases the prevalence and severity of periodontal disease and increases the risk of oral cancer [17]. Lack of information and negative attitudes about good oral health can be another reason for poor health [18]. Oral health extends beyond just toothache and gum pain, from diet to overall health, and can even lead to the risk of death [19]. Currently, oral health and dental care services for prisoners are provided by special medical institutions set up by correctional institutions [20].

The earliest stage of gum disease, called gingivitis, is caused by oral bacteria attacking the soft supporting tissues [21]. Gingivitis does not cause tooth loss [22]. This is because it only infects the soft tissue of the gum, not the alveolar bone [23]. However, if left untreated, it can progress further into a more serious periodontitis condition [24]. When this happens, the soft gum tissue recedes from the bottom of the tooth and leaves spaces called pockets [13–27]. Bacteria from plaque in the mouth can then lodge in these pockets, causing serious infections. Periodontitis is an inflammatory disease that affects the supporting structures of the tooth, both soft and hard tissues [28, 29]. It is caused by specific oral bacteria that induce a host-mediated inflammatory response leading to loss of periodontal attachment [30]. It is a common chronic disorder, estimated to affect, for example, 70% of those 65 years and older [31]. WHO estimates that 80% of adults worldwide have gingivitis [32] and 10–15% have severe periodontitis [31]. The latter condition is the leading cause of tooth loss in adults [33].

The prevalence of periodontal disease among adults in Taiwan is as high as 80.48%, and about 47% of them have serious problems [34]. Among them, the risk of periodontal disease among adults aged 50–64 is 6.7 times that of people aged 18–34 and is also the age group with the highest incidence of periodontal disease [35]. The reasons are mostly a long-term accumulation of oral problems or unconsciousness and neglect of medical care [36, 37]. According to Taiwan’s Ministry of Health and Welfare’s “2015–2016 Annual Adult and Elderly Population Health Survey Report”, the prevalence of periodontal disease in the elderly population over 80 years old is as high as 88.7%, and the prevalence of periodontal disease in adults in 2016 [35]. It is as high as 80.4% in Taiwan, and the risk of periodontal disease in the 50–64 age group is 6.7 times that of the 18–34 age group [35]. The harm of periodontal disease to middle-aged and elderly people is not limited to the oral cavity but may also promote the occurrence or progression of systemic diseases (such as cardiovascular disease, diabetes, etc.), and the impact on health cannot be ignored [38, 39].

According to the 2005 National Health Interview Survey, women pay more attention to oral hygiene than men. The biggest difference is in the rate of brushing teeth before going to bed. 80% of women over 12 years old brush their teeth before going to bed, but less than 70% of men brush their teeth [40]. The reason why women pay more attention to oral health care may not only be that women generally pay more attention to health issues, but also may be because women care more about the smell emitted when talking to others [41]. Men must pay attention to the importance of oral health care, not to mention periodontal health, diseases are related to the frequency and method of brushing teeth [42]. In particular, some systemic diseases such as exacerbation of diabetes, cardiovascular disease, senile pneumonia, and the phenomenon that pregnant women are prone to premature babies and low-birth-weight babies are all related to oral health [43].

In prisons, due to safety considerations, the environmental sanitation conditions are poor, and it is inconvenient for inmates to receive timely medical treatment [44, 45]. Prisoners have a higher burden of mental illness, infectious and chronic diseases, and cognitive impairment [46]. It is important to understand specific oral disease profiles that are particularly relevant to the prison environment and that can be improved. Given the need for equal healthcare for prisoners and the lack of attention paid to them, it is in the public interest to focus on the health of prisoners. Therefore, we estimate the gender-specific prevalence of oral disease in a sample of Taiwanese prisoners.

## Methods

### Study design and setting

We conducted a cross-sectional study design. Information about inmates is shown in a subset of National Health Insurance Research Database (NHIRD) from January 1, 2013, to December 31, 2013. Our study included information from 2013 (January 1 to December 31); therefore, we present results using International Classification of Diseases, Ninth Edition (ICD-9), and International Classification of Diseases, Ninth Edition, Clinical Modification (ICD-9-CM) diagnosis codes. ICD-9 codes (520–529) measure oral, Outcome of salivary gland and jaw diseases. This survey was exempted from written informed consent and was approved by the Ethics Committee of Institutional Review Board of Cheng-Hsin General Hospital (CHGH-IRB: (471)104-07). All procedures followed decision tree for determining whether data science research were carried out according to the standards of our ethics committee and adhered to the tenets of the Declaration of Helsinki. All patients’ information was anonymous.

### Study populations

**Fig. 1:**
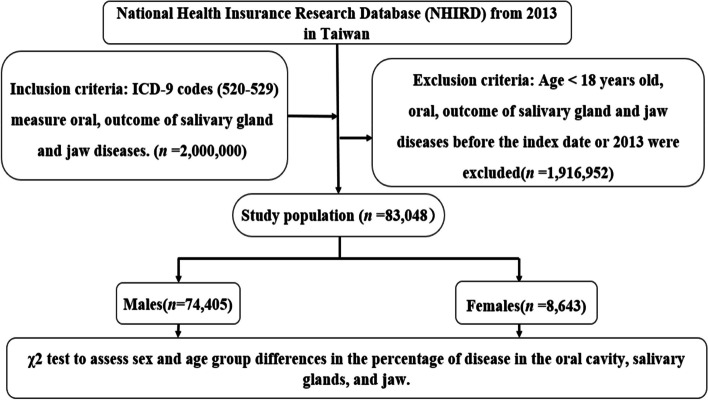
The flowchart of the study sample selection

The study included 83,048 prisoners. Figure 1 shows the study design flow chart for this study. The majority were men (89.59%) and 10.41% were women. We measured outcomes according to ICD-9 codes (520–529) disorders of the oral cavity, salivary glands, and jaw (Table 1). To maintain a strict algorithm, only patients with at least 3 diagnoses in a group were considered disease cases. The ICD is suitable for “all general epidemiological, many health management purposes and clinical applications, including analyzing the general health status of population groups and monitoring the incidence and prevalence of disease and about other variables such as characteristics and conditions of affected individuals, reimbursement, resource allocation, quality, and guidelines” [47].

**Table 1:** ICD 9 diagnosis code – diseases of oral cavity, salivary glands, and jaws-520 to 529

ICD Code	Description
520	Disorders of tooth development and eruption
521	Diseases of hard tissues of teeth
522	Diseases of pulp and periapical tissues
523	Gingival and periodontal diseases
524	Dentofacial anomalies, including malocclusion
525	Other diseases and conditions of the teeth and supporting structures
526	Diseases of the jaws
527	Diseases of the salivary glands
528	Diseases of the oral soft tissues excluding lesions specific to the gingiva and tongue
529	Diseases and other conditions of the tongue

### Data collection tool and procedure

Taiwan’s National Health Insurance (NHI) system covers 99% of the population. NHIRD was developed based on information collected from the Taiwanese population [48]. From 1995 to 2016, NHIRD used the ICD-9-CM. International Classification of Diseases, Tenth Edition (ICD-10) has been used since 2017 [49]. Details of this data source and design are described elsewhere [50].

We have provided more information on how NHI covers dental care services and prison facility services (Additional file 1: Appendix 1). Number of inmates in prisons in Taiwan (by institution) to understand how many people are incarcerated annually in Taiwan (Additional file 1: Appendix 2).

### Statistical analysis

SAS for Windows (version 9.4; SAS Institute Inc., Cary, NC, USA) was used to perform all analysis in this study. The study provides mean and standard deviation (SD) for age. For prevalence, the study provides absolute values and percentages. The study also performed a χ2 test to assess sex and age group differences in the percentage of disease in the oral cavity, salivary glands, and jaw.

## Results

The age of the participants is shown in Table 2. The study included 83,048 prisoners (Male/74,405, Female/8,643). The mean ages of female and male prisoners were 38.65 (SD: 11.79) and 41.50 (SD: 11.43), respectively. The mean ages for females and males with oral cavity, salivary glands, and jaws were 37.14(SD:11.40) and 39.93(SD:11.07), respectively.

**Table 2:** Age and Medicine Service Times (a year) of the participating sample by gender (*n* = 83,048, Taiwan, 2013)

Variables	Total		Diseases of the oral cavity, salivary glands, and jaws	
	Female (*n* = 8,643)	Male (*n* = 74,405)	Female (*n* = 3,128)	Male (*n* = 18,383)
Age (years)				
Mean (standard deviation, SD)	38.65 (11.79)	41.50 (11.43)	37.14 (11.40)	39.93 (11.07)
Range (min-max)	2-88	2-103	2-87	2-103
Medicine Service Times (a year)				
Mean (standard deviation)	18.17 (15.83)	13.27 (13.16)	22.14 (16.61)	17.51 (15.14)
Range (min-max)	1-228	1-369	2-228	2-315

The prevalence of oral cavity, salivary glands, and jaw diseases according to sex is shown in Table 3. The prevalence of oral cavity, salivary glands, and jaw disease among females were 36.19%, while the prevalence in males was 24.70% (*p* < 0.001). Among female prisoners, the top three oral cavity, salivary glands, and jaws diseases were hard tissues of teeth (19.26%), diseases of Gingival and periodontal (16.68%), and diseases of pulp and periapical tissues (4.71%). The top three oral cavity, salivary glands, and jaws diseases in male prisoners were hard tissues of teeth (12.58%), Gingival and periodontal (9.02%), and diseases of the oral soft tissues excluding lesions specific to gingiva and tongue (2.60%).

**Table 3:** Prevalence of oral cavity, salivary glands, and jaw diseases by gender by a survey of the year 2013 claims data from the Taiwan National Health Insurance program (*n* = 83,048, Taiwan, 2013)

Variables	Female			Male		
	*n*	%	mean age (SD)	*n*	%	mean age (SD)
Total prisoners	8,643	10.41	38.65 (11.79)	74,405	89.59	41.50 (11.43)
ICD9_520-529 Diseases of the oral cavity, salivary glands, and jaws	3,128	36.19	37.14 (11.40)	18,383	24.70	39.93 (11.07)
ICD9_520- Disorders of tooth development and eruption	30	0.35	31.20 (12.02)	176	0.24	35.26 (8.68)
ICD9_521- Diseases of hard tissues of teeth	1,665	19.26	35.94 (10.81)	9,361	12.58	38.42 (10.83)
ICD9_522- Diseases of pulp and periapical tissues	407	4.71	36.50 (10.48)	1,985	2.67	39.55 (10.63)
ICD9_523- Gingival and periodontal diseases	1,422	16.68	37.38 (11.24)	6,709	9.02	40.99 (11.22)
ICD9_524-Dentofacial anomalies including malocclusion	21	0.24	32.29 (9.74)	94	0.13	38.95 (11.17)
ICD9_525- Other diseases and conditions of the teeth and supporting structures	393	4.55	37,22 (8.80)	863	1.16	42.33 (10.68)
ICD9_526- Diseases of the jaws	2	0.02	40.00 (14.14)	32	0.04	46.47 (11.25)
ICD9_527- Diseases of the salivary glands	10	0.12	39.00 (7.16)	46	0.06	42.76 (11.62)
ICD9_528- Diseases of the oral soft tissues excluding lesions specific for gingiva and tongue	319	3.70	39.05 (12.01)	1,932	2.60	41.93 (11.70)
ICD9_529- Diseases and other conditions of the tongue	0	-	-	29	0.04	48.14 (10.88)

The prevalence of the most frequent oral cavity, salivary glands, and jaw diseases by sex are shown in Table 4. The diseases with the highest prevalence were hard tissues of teeth diseases (13.28%), gingival and periodontal diseases (9.79%), and diseases of pulp and periapical tissues (2.88%). 25.90% of prisoners had oral cavities, salivary glands, and jaw diseases. There was a significant difference between females and males in the prevalence of oral cavity diseases except for diseases of dentofacial anomalies including malocclusion, salivary glands, jaws, and other conditions of the tongue.

**Table 4:** The prevalence of oral cavity, salivary glands, and jaw diseases among prisoners by gender (*n* = 83,048, Taiwan, 2013)

Variables	Total	Female		Male		*p* for χ^2^ test
	%	*n*	%	*n*	%	
Total prisoners		8,643	10.41	74,405	89.59	
ICD9_520-529 Diseases of the oral cavity, salivary glands, and jaws	25.90	3,128	36.19	18,383	24.70	<0.001
ICD9_520- Disorders of tooth development and eruption	0.25	30	0.35	176	0.24	0.05
ICD9_521- Diseases of hard tissues of teeth	13.28	1,665	19.26	9,361	12.58	<0.001
ICD9_522- Diseases of pulp and periapical tissues	2.88	407	4.71	1,985	2.67	<0.001
ICD9_523- Gingival and periodontal diseases	9.79	1,422	16.68	6,709	9.02	<0.001
ICD9_524-Dentofacial anomalies including malocclusion	0.14	21	0.24	94	0.13	0.006
ICD9_525- Other diseases and conditions of the teeth and supporting structures	1.51	393	4.55	863	1.16	<0.001
ICD9_526- Diseases of the jaws	0.04	2	0.02	32	0.04	0.180
ICD9_527- Diseases of the salivary glands	0.07	10	0.12	46	0.06	0.068
ICD9_528- Diseases of the oral soft tissues excluding lesions specific for gingiva and tongue	2.71	319	3.69	1932	2.60	<0.001
ICD9_529- Diseases and other conditions of the tongue	0.03	0	-	29	0.04	0.041

The prevalence of oral cavity, salivary glands, and jaw diseases according to age is shown in Table 5. The prevalence of oral cavity, salivary glands, and jaw disease among prisoners who were ≤ 40 was 29.02%, while the prevalence who were > 40 was 22.59%. Among prisoners who were ≤ 40, the top three oral cavity, salivary glands, and jaws diseases were hard tissues of teeth diseases (16.39%), gingival and periodontal diseases (10.33%), and pulp and periapical tissues diseases (3.45%). The top three oral cavity, salivary glands, and jaw diseases in prisoners who were > 40 were hard tissues of teeth diseases (9.98%), gingival and periodontal diseases (9.22%), and the oral soft tissues excluding lesions specific for gingiva and tongue diseases (2.82%).

**Table 5:** Prevalence of oral cavity, salivary glands, and jaw diseases by age group bya survey of the year 2013 claims data from the Taiwan National Health Insurance program (*n* = 83,048, Taiwan, 2013)

Variables	≤40			>40		
	*n*	%	mean age (SD)	*n*	%	mean age (SD)
Total prisoners	42,684	51.40	32.30 (6.16)	40,364	48.60	50.62 (7.71)
ICD9_520-529 Diseases of the oral cavity, salivary glands, and jaws	12,389	29.02	31.94 (6.26)	9,122	22.59	49.83 (7.48)
ICD9_520- Disorders of tooth development and eruption	161	0.38	30.92 (5.81)	45	0.11	48.07 (6.80)
ICD9_521- Diseases of hard tissues of teeth	6,998	16.39	31.59 (6.35)	4,028	9.98	49.26 (7.39)
ICD9_522- Diseases of pulp and periapical tissues	1,473	3.45	32.38 (5.78)	919	2.28	49.70 (7.59)
ICD9_523- Gingival and periodontal diseases	4,410	10.33	32.13 (6.16)	3,721	9.22	50.11 (7.68)
ICD9_524-Dentofacial anomalies including malocclusion	75	0.18	30.99 (5.97)	40	0.10	50.38 (6.83)
ICD9_525- Other diseases and conditions of the teeth and supporting structures	712	1.67	33.43 (4.89)	544	1.35	50.29 (7.55)
ICD9_526- Diseases of the jaws	9	0.02	32.78 (5.43)	25	0.06	50.88 (8.65)
ICD9_527- Diseases of the salivary glands	25	0.06	32.72 (6.15)	31	0.08	49.65 (7.71)
ICD9_528- Diseases of the oral soft tissues excluding lesions specific for gingiva and tongue	1,112	2.61	32.08 (6.65)	1,139	2.82	50.73 (7.70)
ICD9_529- Diseases and other conditions of the tongue	7	0.02	33.43 (7.50)	22	0.05	52.82 (6.84)

The prevalence of the most frequent oral cavity, salivary glands, and jaw diseases by age group are shown in Table 6. There was a significant difference between ≤ 40 and > 40 in the prevalence of almost all categories of the oral cavity, salivary glands, and jaws, except for the salivary gland’s diseases, the oral soft tissues excluding lesions specific for gingiva and tongue diseases, and diseases and other conditions of the tongue.

**Table 6:** The prevalence of oral cavity, salivary glands, and jaw diseases among prisoners by age group (*n* = 83,048, Taiwan, 2013)

Variables	Total	≤40		>40		*p* for χ^2^ test
	%	*n*	%	*n*	%	
Total prisoners		42,684	51.40	40,364	48.60	
ICD9_520-529 Diseases of the oral cavity, salivary glands, and jaws	25.90	12,389	29.02	9,122	22.59	<0.001
ICD9_520- Disorders of tooth development and eruption	0.25	161	0.38	45	0.11	<0.001
ICD9_521- Diseases of hard tissues of teeth	13.28	6,998	16.39	4,028	9.98	<0.001
ICD9_522- Diseases of pulp and periapical tissues	2.88	1,473	3.45	919	2.28	<0.001
ICD9_523- Gingival and periodontal diseases	9.79	4,410	10.33	3,721	9.22	<0.001
ICD9_524-Dentofacial anomalies including malocclusion	0.14	75	0.18	40	0.10	0.003
ICD9_525- Other diseases and conditions of the teeth and supporting structures	1.51	712	1.67	544	1.35	<.001
ICD9_526- Diseases of the jaws	0.04	9	0.02	25	0.06	0.004
ICD9_527- Diseases of the salivary glands	0.07	25	0.06	31	0.08	0.312
ICD9_528- Diseases of the oral soft tissues excluding lesions specific for gingiva and tongue	2.71	1,112	2.61	1,139	2.82	0.055
ICD9_529- Diseases and other conditions of the tongue	0.03	7	0.02	22	0.05	0.003

Additional file 1: Appendix 3 shows the prevalence of Diseases of the oral cavity, salivary glands, and jaws by age group by a survey of year 2013 claims data from the Taiwan National Health Insurance program. Additional file 1: Appendix 4 shows the prevalence of Diseases of the oral cavity, salivary glands, and jaws among prisoners by age group.

## Discussions

### Clinical implications

Based on our findings in Taiwanese prisons, where there are more men than women, dentists should consider more products suitable for male prisoners. In prisons, healthcare professionals should be aware of the higher prevalence of oral, salivary gland, and jawbone disease in female prisoners with oral disease compared with male prisoners and provide care to prisoners with chronic conditions such as oral disease.

According to statistics from the National Health Administration of the Ministry of Health and Welfare, as many as 90% of domestic adults suffer from periodontal disease [25]. Clinical statistics show that people who visit the doctor because of periodontal disease or other symptoms (such as tooth cleaning, dental implants, and regular check-ups) are diagnosed with periodontal disease [51]. Physicians informed of precursor symptoms or exposure to the risk of periodontal disease [52]. Among oral diseases, periodontal disease has the highest incidence and is easily overlooked [53]. The main cause of disease is bacterial infection, and the treatment method is mainly to remove the bacteria in the lesion [54]. It is not uncommon for periodontal disease to cause systemic diseases such as myocarditis, osteoporosis, respiratory disease, hepatitis, diabetes, and stroke if it is not properly treated [55]. A study conducted big data analysis on 160,000 patients and found that the number of periodontal diseases in all age groups is on the rise [56]. It is worth noting that the 31–40 age group is the crown of the ethnic group, the proportion of young adults suffering from periodontal disease is increasing year by year [57]. The main reason is that people have insufficient awareness of periodontal disease [58]. Because periodontal disease has only slight tooth sensitivity in the early stage and bleeding when brushing or flossing, not many people seek medical treatment, which delays the golden opportunity for treatment [59].

Although prisoners may be easily punished, they have equal access to medical care [60]. Efforts to improve public health and reduce health inequalities should include prisons, an effort by countries to leave no one behind, achieve universal health coverage, and achieve the United Nations Sustainable Development Goals [61]. ‎WHO recommends reducing any “avoidable or unfair” health disparities, stating that prisoners have the right to equal access to medical care [62]. Therefore, this study was carried out to understand the oral, salivary gland, and jaw disease profiles of prisoners. Previously, we conducted a study to understand the prevalence of skin diseases in prisoners, which could be part of a series of studies [63]. To the best of our knowledge, this is the first study describing oral disease in Taiwanese prisoners. The main finding was that the prevalence of oral diseases was higher in prisoners (25.90%) than in the general population (12.4%) [64]. The results also showed that female prisoners had a higher prevalence (36.19%) of oral, salivary gland, and jaw diseases than male prisoners (24.70%).

The prevalence of oral, salivary gland, and jaw disorders in our study was estimated at 25.90%, suggesting that staff working in prisons should be concerned about the exact number of prisoners with oral, salivary gland, and jaw disorders. One study found that more than half of patients with oral disease believed that detention was directly related to oral disease [65]. A recent systematic review of 21 studies showed that the prevalence of oral disease is higher in prison populations than in the general population [66]. Previous research has shown that oral disease is common among detainees. This prevalence may be due to the absence of oral hygiene equipment in detention facilities and the lack of oral hygiene knowledge or care among inmates, leading to the development of dental caries and periodontal disease [67]. Several studies have shown the importance of prison dental services, which can provide better access to care and increase prisoners’ awareness of the importance of proper hygiene [68, 69].

A systematic review supported an association between prison population density and infectious disease despite the poor quality of the included studies [70]. Overcrowding and poor prison conditions are also associated with the mental state of prisoners [71]. The health problems of prisoners are not only a problem of the prison itself; it is also a social problem, because more than 95% of prisoners will eventually return to normal life, and their health problems can become a burden on the community and even society [72].

The prevalence of oral, salivary gland, and jaw diseases was higher in female prisoners than in male prisoners in Taiwan. Women’s oral microbiota differs somewhat from men’s, and hormonal changes may be an important factor [73]. For example, changes in women’s hormonal levels during the menstrual cycle may affect the oral environment, which in turn affects the population and composition of oral bacteria [74]. Menopause and menopause in women cause a series of changes in the mouth because falling estrogen levels reduce the amount of saliva in the mouth [75]. Saliva is protective, it cleans teeth and washes away cavity-causing bacteria, and it helps neutralize acidity in the mouth, so less saliva can lead to dry mouth, increasing the risk of tooth decay [76]. Lower estrogen puts you at higher risk for bone loss, and bone loss in the jaw can lead to gum recession, which can expose teeth and increase the risk of cavities [77]. The secretion of male salivary glands may decrease with age, this is because as we age, the function of our salivary glands may be affected, resulting in reduced saliva production [78]. In the general population, sex differences exist in the prevalence of diseases of the oral cavity, salivary glands, and jaw due to body structure and function, immune system, and sex hormones in men and women [75]. Sex differences make it important to produce and prepare male-specific oral products for men [79].

Prisoner’s health problems are not only a prisoner’s problem; it is also a social problem as more than 95% of prisoners eventually return to normal life and their health problems can become a burden to the community and even society. Therefore, relevant units are strengthening oral health issues in Taiwan’s prisons. Without oral care, there is no access to medical care. National health care must include oral health services for prison inmates to protect the oral health of prisoners.

### Methodological considerations

An obvious strength of this study is the large sample size, which allowed us to obtain a profile of oral, salivary gland, and jaw diseases in Taiwanese prisoners. In addition, we included information from Taiwan rather than just one prison. Results are measured according to a harmonized ICD code; thus, measurement bias is not only largely avoided, but the comparability of our results was improved.

In addition, it must be acknowledged that this study has some limitations. Firstly, this was a descriptive cross-sectional study; therefore, we were unable to identify any risk factors or draw any causality from the analysis. Future longitudinal studies are needed to determine potential oral, salivary gland, and jaw disease exposures. Secondly, all participants were from Taiwanese prisons; therefore, generalizability to other places or ethnicities may be limited. Third, NHIRD only records the gender, age, and symptoms of common medical diseases of prisoners, we must consult other criminal databases of the Ministry of Justice for information on length of imprisonment, type of crime, detention facility, Taiwanese locals and non-locals, and education level. Fourth, if people don’t have access to dental services, they won’t be able to detect oral health problems. This study may underestimate the number of prisoners who do not seek medical treatment for oral problems. Fifth, although the chi-squared test applies an approximation assuming the sample is large, while the Fisher’s exact test runs an exact procedure especially for small-sized samples. Chi-square tests are highly influenced by sample size. Given the size of the sample, almost any small difference will be statistically significant [80, 81]. Finally, with a short 1-year follow-up, this study was unable to provide trends in oral, salivary gland, and jaw disease among Taiwanese prisoners.

## Conclusions

In this study, we observed a higher prevalence of oral, salivary gland, and jaw diseases in Taiwanese prisoners than in the general population. We also found female prisoners had a higher prevalence of oral, salivary gland, and jaw diseases than male prisoners. These findings underscore the importance of identifying and treating oral disease in Taiwanese prisons. In addition, it serves as a reminder to strengthen health equality in Taiwanese prisons. Access to healthcare cannot be achieved without oral healthcare. National health care must include oral health services to safeguard the oral health of prisoners. Future studies should include non-prisoners to further assess how oral health differs between people in prison versus those not in prison.

### Supplementary Information

**Additional file 1: Appendix 1. **NHI covers dental care services and prison facility services. **Appendix 2. **Number of inmates in prisons in Taiwan (by institution) to understand how many people are incarcerated annually in Taiwan.** Appendix 3. **The prevalence of diseases of the oral cavity, salivary glands, and jaws by age group by a survey of year 2013 claims data from the Taiwan National Health Insurance program.** Appendix 4. **The prevalence of diseases of the oral cavity, salivary glands, and jaws among prisoners by age group.

## Data Availability

All data generated or analyzed during this study are included in this published article.

## Abbreviations

ICD-9-CM: International Classification of Diseases, Ninth Edition
ICD-9-CM: International Classification of Diseases, Ninth Edition, Clinical Modification
ICD-10: International Classification of Diseases, Tenth Edition
NHI: National Health Insurance
NHIRD: National Health Insurance Research Database
WHO: World Health Organization
