# Supplementary material for: Gender differences in oral health among prisoners: a cross-sectional study from Taiwan
Source: BMC Oral Health. 2023 Nov 21;23:900. doi: 10.1186/s12903-023-03598-4 (PMC10662814; doi:10.1186/s12903-023-03598-4)
Supplement: Supplementary file 1 — Additional file 1: Appendix 1. NHI covers dental care services and prison facility services. Appendix 2. Number of inmates in prisons in Taiwan (by institution) to understand how many people are incarcerated annually in Taiwan. Appendix 3. The prevalence of diseases of the oral cavity, salivary glands, and jaws by age group by a survey of year 2013 claims data from the Taiwan National Health Insurance program. Appendix 4. The prevalence of diseases of the oral cavity, salivary glands, and jaws among prisoners by age group. [file 12903_2023_3598_MOESM1_ESM.doc]

**Appendix legends**

Appendix 1. NHI covers dental care services and prison facility services.

Appendix 2. Number of inmates in prisons in Taiwan (by institution) to understand how many people are incarcerated annually in Taiwan.

Appendix 3. The prevalence of diseases of the oral cavity, salivary glands, and jaws by age group by a survey of year 2013 claims data from the Taiwan National Health Insurance program.

Appendix 4. The prevalence of diseases of the oral cavity, salivary glands, and jaws among prisoners by age group.

**Appendix 1:**

**Instructions for Operation of National Health Insurance in Providing Medical Services in Correctional Institutions**

The environment of medical facilities in correctional institutions is different from that of medical institutions, and the recipients are also subject to restrictions on personal freedom. In order to ensure the smooth provision of health insurance and medical care in correctional institutions, while taking into account the management needs of correctional institutions, safeguarding the safety of medical personnel and the medical rights and interests of inmates, this notice is formulated.

1. Medical treatment medical treatment system, information equipment, etc. to facilitate smooth medical treatment process.

2. The physician's medical treatment of the inmates shall be based on their medical needs and professional judgment. The issuance of relevant certificates should also adhere to the medical profession and be annotated according to the diagnosis results. It should not be accompanied by words indicating the recommended targets of containment, or words related to non -medical diagnosis. If the inmates make the following requests, they should be rejected, and the staff of the health department of the correctional institution should be notified of the situation.

(1) Increasing the dosage of medicines (this situation is common in psychosomatic departments). (2) The use of specific drugs or health materials, such as requiring a physical and mental doctor to prescribe Class 3 or 4 controlled drugs. (3) Issue a referral order and even require the doctor to specify the referral time and hospital so that he or she can anticipate the time and place of receiving medical treatment at the hospital. (4) Use self-financed items that are not necessary for treatment, such as patches, stomach powder and other proprietary medicines. (5) Use injections that are not necessary for treatment. (6) Non-essential surgical treatment (such as lipoma, first to second degree hemorrhoids) requires hospitalization by a foreign surgeon. (7) Move into units designated by correctional institutions, such as nursing homes, observation rooms, isolation rooms, etc. attached to the institution.

3. Physicians may ask inmates to provide medication records of long-term use of prescribed drugs before internment, as needed for diagnosis and treatment. 1. According to Article 3, Item 1 of the Measures for the Administration of Medical Treatment of Persons Insured by the National Health Insurance in Correctional Institutions, in the event of illness, injury or childbirth, the inmates shall have priority over the medical treatment in the correctional institutions. Therefore, unless proper diagnosis and treatment, examination (inspection) cannot be performed in the correctional institution or there is a medical emergency, it should be avoided to recommend that the confinement be transferred to an insurance medical service institution for medical treatment. For example, non-urgent specimens that can be collected and submitted for testing by medical personnel of insurance medical service institutions in correctional institutions should be avoided to make the inmates guard against foreign doctors.

4. Insurance medical service institutions may provide medical facilities related to outpatient diagnosis and treatment and inspection (examination) in the correctional institution according to the results of consultation with the correctional institution, so as to facilitate the diagnosis and treatment of doctors, improve the timeliness of inpatient medical treatment and reduce the need for outpatient care frequency.

5. Request the correctional institution or insurance medical service agency in charge to regularly maintain the diagnostic and treatment equipment (facilities) in the correctional institution, if Medical treatment system, information equipment, etc. to facilitate smooth medical treatment process.

6. If the correctional institution has an emergency patient, it must be admitted to the outpatient department of the institution on the same day. You can contact the insurance medical service or ask the correctional institution to provide records of the usual sleep conditions or emotional expressions of patients attending the psychosomatic department.

7. Correctional institutions should cooperate with the following matters before receiving medical treatment to facilitate the smooth operation of the medical treatment.

(1) Take the patients to the waiting place before opening the clinic to avoid delaying the consultation and prolonging the outpatient hours. (2) Confirm that the inmates carry their health insurance cards for medical treatment. (3) Differentiate acute and severe patients and ask doctors to see them first. (4) Provide information on the contagious diseases of the inmates for reference by medical personnel in diagnosis and treatment, so as to ensure the safety of the inpatients' medication and protect the medical staff.

8. When medical personnel provide medical services, they should avoid discrimination, such as refusing treatment for patients with notifiable infectious diseases.

9. When diagnosing and treating the host's disease and requiring self-pay items or using special materials, the medical staff should explain the reason and amount of the self-pay, and then ask the host to fill in a self-pay consent form and issue a receipt to reduce disputes.

10. After the insurance medical service agency provides medical services to the detainee in the correctional institution, it should transmit the detainee’s medical records to the SFTP host designated by the Ministry of Justice for the correctional institution to import. The upload time is in principle within two working days from the date of consultation.

11. Medical personnel should provide medical services according to the scheduled time. If there is any change, they should provide medical service plans for insured persons admitted to correctional institutions in accordance with the National Health Insurance and report to the insurance divisional business group for approval and approval. Assistant Correctional Institution. If the correctional institution plans to suspend clinics, it should also notify the insurance medical service institution in advance in accordance with the aforementioned regulations.

12. The correctional institution may assist in deducting the medical expenses incurred by the institutionalized object from the custody fund and labor compensation of the institutionalized object and disbursing them to the insured medical service provider on a monthly basis. Correctional institutions should fulfill their responsibilities for deductions and collections during the period of detention.

13. If the correctional institution needs relevant information on the inmate’s incarceration and transfer to an insurance medical service institution for medical treatment, please apply for a diagnosis certificate or a copy of the medical record in accordance with the regulations.

2. Management

1. When there are more than 3 days (inclusive) of consecutive holidays (for example: Spring Festival), the insurance medical service institution is required to provide at least 1 outpatient clinic every 3 days in the correctional institution or adjust the number of clinics according to actual needs. The consultation time should be prepared in advance Coordinate with correctional agencies. In order to facilitate advance arrangements by insurance medical service providers, correctional institutions are requested to plan in advance the opening dates during consecutive holidays before the start of the year (it is recommended to avoid Sundays and the first and second days of the Lunar New Year as a rule) and notify the insurance medical service Institutions and the divisional business groups of insurers under their jurisdiction. Continuous holidays are defined in accordance with the office calendar of the administrative agency.

2. Correctional institutions should notify the insurance medical service agency in advance when providing medical care to a subject under detention. However, due to safety considerations, the correctional institution may not include the subject’s personal information. After receiving the notification, insurance medical service institutions should assist in making arrangements according to their standards to maintain the safety of nursing care. The inmates and correctional institution personnel should abide by the regulations of insurance medical service institutions to facilitate medical operations. 8. When medical personnel provide medical services, they should avoid discrimination, such as refusing treatment for patients with notifiable infectious diseases.

3. When medical personnel from insurance medical service institutions provide medical services to correctional institutions, they should avoid bringing items unrelated to medical services. Before entering the guarded area, you should put your personal belongings, such as mobile phones and money, in the storage cabinet provided by the correctional institution, and abide by the relevant regulations for entering the correctional institution.

4. Medical personnel are not allowed to bring any items into and out of the correctional institutions for the inmates, such as food, cash, cigarettes, Betel nuts, lighters, letters, notes, medicines, mobile phones, etc. to avoid being subject to relevant penalties.

5. Medical personnel are not allowed to introduce products that are not related to the illness to the shelter recipients, or their family members, relatives and friends. The medicines used by the inmates should be mainly items covered by health insurance. If there is a need to use health food or other patent medicines, the correctional agency can handle the purchase in accordance with existing regulations.

6. The time and place of the detainee’s medical outpatient care, hospitalization or examination (examination) shall be handled by the correctional institution in accordance with the provisions of Article 3, Item 2 of the Regulations on the Medical Treatment Management of Persons Confined to the Correctional Institution under the National Health Insurance. It is not allowed to designate on its own, and the personnel of the correctional institution and the medical staff are not allowed to inform the inmates of the time and location of the foreign doctor in advance.

7. When medical personnel provide medical services in correctional institutions, they should provide medical services within designated areas and are not allowed to wander to other workshops or parishes without authorization. Correctional institutions shall not require medical personnel to provide medical services in other areas alone without the accompaniment of correctional institution personnel.

8. When medical staff find that the inmate needs enhanced counseling and care, they should notify the correctional agency or record it so that the correctional agency can provide counseling and necessary assistance or make a referral.

9. When correctional institutions provide medical services to medical personnel, they should cooperate with the following matters to maintain the safety of medical personnel and avoid medical disputes: (1) Assist in on-site safety surveillance. (2) To prevent the inmates from bringing unsafe items to seek medical treatment. (3) When female detainees undergo examination (investigation), they should be accompanied by personnel from the female correctional institution.

10. When an inmate complains about a medical dispute, the correctional institution shall assist in the investigation and handling.

11. In the event of natural disasters such as typhoons during the outpatient period of the correctional institution, the clinic will be closed according to the announcement of the suspension of work and class by the county and city governments.

12. If the inmate has medical needs and the insurance medical service institution assists in applying for the items that should be reviewed beforehand for the national health insurance, the insurance medical service institution shall notify the personnel of the correction agency of the application items and the pre-examination results.

**Participation and Interaction**

1. Medical personnel should uphold a professional image when providing medical services, maintain an appropriate distance from the recipients, and avoid contact with the recipients. Containment subjects develop personal feelings or have special sympathy and emotional responses to the case.

2. When providing medical services, medical personnel should avoid discussing issues unrelated to the condition of the recipients, such as political party affiliation, 9. When correctional institutions provide medical services to medical personnel, they should cooperate with the following matters to maintain the safety of medical personnel and avoid medical disputes: (1) Assist in on-site safety surveillance. (2) To prevent the inmates from bringing unsafe items to seek medical treatment. (3) When female detainees undergo examination (investigation), they should be accompanied by personnel from the female correctional institution. Current events, religious beliefs, etc.

3. When the inmates report to the medical staff about life adaptation or other opinions that have nothing to do with the disease, they are asked to report to the correctional staff reaction.

4. When medical personnel provide medical services, if they encounter unreasonable demands or safety concerns from a subject, or verbal offense or physical harassment from a subject, they should immediately notify on-site security personnel to deal with the situation.

5. Medical personnel should not be asked by others to contact relatives and friends on behalf of the recipient, or to send messages to the recipient.

6. Medical personnel are not allowed to lend, use, or invite banquets and entertainment with money or property to the interns or their relatives and friends.

7. Medical personnel should respect the personality of the asylum seekers and pay attention to the protection of their personal data. With the permission of the person concerned, no relevant information about the correctional institution or the inmates shall be released to the outside world.

8. Medical personnel are not allowed to leave the telephone numbers, addresses and other personal information of individuals or service agencies to the inmates. Correctional institutions know the personal information of medical personnel for business purposes and must not leak it.

9. Correction institutions should provide dedicated telephones (extensions), or create a contact mechanism, so that insurance medical service institutions may encounter In emergency situations such as critical patients, the medical staff can be contacted immediately.

10. Please dress neatly and dignifiedly for medical personnel.

**Appendix 2:**

| Appendix 2. **Number of inmates in prisons in Taiwan (by institution)** | | | | | | | | | | |
| --- | --- | --- | --- | --- | --- | --- | --- | --- | --- | --- |
|  | Republic of China (Taiwan) | | | | January-July 112 | | | | | Unit: person |
| Organization type | Capacity | | | | Number of people released from prison | | | | | Number of people in prison at the end of the period |
|  | total | Number of people in prison at the end of last year | Number of people in prison |  |  | Actual number of people released from prison | | | |  |
|  |  |  |  | Number of new prisoners |  | count | execution | Released  from prison upon completion of execution | parole |  |
| Total | 82,949 | 49,720 | 33,229 | 17,972 | 33,192 | 18,149 | - | 13,441 | 4,708 | 49,757 |
| Taipei prison | 11,478 | 5,914 | 5,564 | 3,225 | 5,473 | 2,774 | - | 2,334 | 440 | 6,005 |
| Taoyuan Prison | 3,785 | 1,090 | 2,695 | 1,661 | 2,616 | 779 | - | 681 | 98 | 1,169 |
| Taoyuan Women's Prison | 1,682 | 1,034 | 648 | 470 | 597 | 425 | - | 303 | 122 | 1,085 |
| Bade Foreign Service Prison | 414 | 297 | 117 | 3 | 118 | 103 | - | 17 | 86 | 296 |
| Hsinchu Prison | 3,517 | 1,846 | 1,671 | 935 | 1,563 | 554 | - | 427 | 127 | 1,954 |
| Taichung Prison | 12,710 | 7,046 | 5,664 | 3,402 | 5,708 | 2,898 | - | 2,102 | 796 | 7,002 |
| Taichung Women's Prison | 1,675 | 1,210 | 465 | 289 | 532 | 380 | - | 198 | 182 | 1,143 |
| Changhua Prison | 3,771 | 2,411 | 1,360 | 756 | 1,483 | 927 | - | 701 | 226 | 2,288 |
| Yunlin Prison | 1,388 | 968 | 420 | 9 | 414 | 271 | - | 217 | 54 | 974 |
| Yunlin No. 2 Prison | 3,718 | 2,167 | 1,551 | 521 | 1,245 | 600 | - | 455 | 145 | 2,473 |
| Chiayi Prison | 4,152 | 2,829 | 1,323 | 627 | 1,468 | 987 | - | 739 | 248 | 2,684 |
| Tainan Prison | 5,735 | 3,365 | 2,370 | 1,252 | 2,093 | 1,290 | - | 1,064 | 226 | 3,642 |
| Tainan No. 2 Prison | 1,423 | 902 | 521 | 20 | 585 | 382 | - | 285 | 97 | 838 |
| Mingde Foreign Service Prison | 599 | 411 | 188 | 7 | 180 | 148 | - | 22 | 126 | 419 |
| kaohsiung prison | 2,555 | 2,189 | 366 | 96 | 437 | 237 | - | 106 | 131 | 2,118 |
| Kaohsiung No. 2 Prison | 3,959 | 1,761 | 2,198 | 1,719 | 2,140 | 1,424 | - | 1,280 | 144 | 1,819 |
| Kaohsiung Women's Prison | 1,496 | 1,117 | 379 | 272 | 433 | 330 | - | 182 | 148 | 1,063 |
| Pingtung Prison | 4,023 | 2,782 | 1,241 | 875 | 1,295 | 966 | - | 723 | 243 | 2,728 |
| Taitung Prison | 4,315 | 3,321 | 994 | 356 | 1,254 | 839 | - | 501 | 338 | 3,061 |
| Hualien Prison | 2,344 | 1,674 | 670 | 399 | 691 | 484 | - | 354 | 130 | 1,653 |
| Ziqiang Foreign Service Prison | 471 | 365 | 106 | 4 | 144 | 113 | - | 10 | 103 | 327 |
| Yilan Prison | 4,869 | 3,282 | 1,587 | 622 | 1,606 | 688 | - | 365 | 323 | 3,263 |
| Keelung Prison | 1,065 | 363 | 702 | 296 | 705 | 294 | - | 270 | 24 | 360 |
| Penghu Prison | 1,300 | 1,054 | 246 | 63 | 257 | 162 | - | 50 | 112 | 1,043 |
| Green Island Prison | 80 | 63 | 17 | 1 | 15 | 9 | - | 4 | 5 | 65 |
| Mingyang Middle School | 185 | 114 | 71 | 13 | 61 | 22 | - | 3 | 19 | 124 |
| Golden Gate Prison | 240 | 145 | 95 | 79 | 79 | 63 | - | 48 | 15 | 161 |
| Note: Prison statistics in this table include prison data. | | | | | | | | | | |

**Appendix 3:**  Prevalence of Diseases of oral cavity, salivary glands, and jaws by age group by a survey of year 2013 claims data from the Taiwan National Health Insurance program

(*n*=83,048, Taiwan, 2013).

|  | <30 | | | | 30-49 | | | | 50-64 | | | | | ≧65 | | | | |  |
| --- | --- | --- | --- | --- | --- | --- | --- | --- | --- | --- | --- | --- | --- | --- | --- | --- | --- | --- | --- |
|  |  | *n* | % | mean age (S.D.) |  | *n* | % | mean age (S.D.) | |  | *n* | % | mean age (S.D.) | |  | *n* | % | mean age (S.D.) | |
| **Total prisoners** |  | 12095 | 14.56 | 24.11(4.21) |  | 51449 | 61.95 | 39.26(5.31) | |  | 17289 | 20.82 | 55.28(4.00) | |  | 2215 | 2.67 | 69.84(5.36) | |
| **ICD9_520-529** |  | 3797 | 31.39 | 23.99(4.11) |  | 13726 | 26.67 | 38.85(5.23) | |  | 3560 | 20.59 | 55.08(3.96) | |  | 428 | 19.32 | 69.65(5.14) | |
| ICD9_520 |  | 64 | 0.53 | 25.36(4.28) |  | 128 | 0.25 | 36.90(5.10) | |  | 12 | 0.07 | 55.33(3.45) | |  | 2 | 0.09 | 65.50(0.71) | |
| ICD9_521 |  | 2289 | 18.93 | 23.85(4.01) |  | 7125 | 13.85 | 38.42(5.14) | |  | 1443 | 8.35 | 54.94(3.95) | |  | 169 | 7.63 | 70.10(5.52) | |
| ICD9_522 |  | 433 | 3.58 | 25.03(3.94) |  | 1562 | 3.04 | 38.44(5.14) | |  | 355 | 2.05 | 54.92(3.82) | |  | 42 | 1.90 | 70.88(5.62) | |
| ICD9_523 |  | 1346 | 11.13 | 24.26(3.83) |  | 5097 | 9.91 | 39.16(5.23) | |  | 1494 | 8.64 | 55.10(3.91) | |  | 194 | 8.76 | 70.05(5.49) | |
| ICD9_524 |  | 28 | 0.23 | 24.57(3.59) |  | 68 | 0.13 | 38.06(5.67) | |  | 18 | 0.10 | 55.11(4.59) | |  | 1 | 0.05 | - | |
| ICD9_525 |  | 148 | 1.22 | 25.81(3.12) |  | 856 | 1.66 | 38.56(5.20) | |  | 229 | 1.32 | 55.59(3.94) | |  | 23 | 1.04 | 69.91(4.37) | |
| ICD9_526 |  | 2 | 0.02 | 25(5.66) |  | 21 | 0.04 | 41,52(5.36) | |  | 8 | 0.05 | 55.13(4.76) | |  | 3 | 0.14 | 68(2.65) | |
| ICD9_527 |  | 4 | 0.03 | 20.25(2.63) |  | 38 | 0.07 | 39.18(5.29) | |  | 12 | 0.07 | 54.08(4.25) | |  | 2 | 0.09 | 69.00(1.41) | |
| ICD9_528 |  | 306 | 2.53 | 22.88(4.46) |  | 1384 | 2.69 | 39.36(5.28) | |  | 493 | 2.85 | 55.37(4.06) | |  | 68 | 3.07 | 68.94(4.22) | |
| ICD9_529 |  | 1 | 0.01 | - |  | 14 | 0.03 | 41.57(5.32) | |  | 12 | 0.07 | 55.58(3.58) | |  | 2 | 0.09 | 65.00(0.00) | |

**Appendix 4:** The prevalence of Diseases of oral cavity, salivary glands, and jaws among prisoners by age group (*n*=83,048, Taiwan, 2013)

|  |  | Total |  | <30 | |  | 30-49 | |  | | 50-64 | |  | ≧65 | | *p* for *X2* test |
| --- | --- | --- | --- | --- | --- | --- | --- | --- | --- | --- | --- | --- | --- | --- | --- | --- |
|  |  | % |  | *n* | % |  | *n* | % |  | *n* | | % |  | *n* | % |  |
| **Total prisoners** |  |  |  | 12095 | 14.56 |  | 51449 | 61.95 |  | 17289 | | 20.82 |  | 2215 | 2.67 |  |
| **ICD9_520-529** |  | 25.90 |  | 3797 | 16.53 |  | 13726 | 4.29 |  | 3560 | | 4.57 |  | 428 | 0.52 | <.0001 |
| ICD9_520 |  | 0.25 |  | 64 | 0.08 |  | 128 | 0.15 |  | 12 | | 0.01 |  | 2 | <0.01 | <.0001 |
| ICD9_521 |  | 13.28 |  | 2289 | 2.76 |  | 7125 | 8.58 |  | 1443 | | 1.74 |  | 169 | 0.20 | <.0001 |
| ICD9_522 |  | 2.88 |  | 433 | 0.52 |  | 1562 | 1.88 |  | 355 | | 0.43 |  | 42 | 0.05 | <.0001 |
| ICD9_523 |  | 9.79 |  | 1346 | 1.62 |  | 5097 | 6.14 |  | 1494 | | 1.80 |  | 194 | 0.23 | <.0001 |
| ICD9_524 |  | 0.14 |  | 28 | 0.03 |  | 68 | 0.08 |  | 18 | | 0.02 |  | 1 | <0.01 | 0.0142 |
| ICD9_525 |  | 1.51 |  | 148 | 0.18 |  | 856 | 1.03 |  | 229 | | 0.28 |  | 23 | 0.03 | <.0001 |
| ICD9_526 |  | 0.04 |  | 2 | <0.01 |  | 21 | 0.03 |  | 8 | | 0.01 |  | 3 | <0.01 | 0.0906 |
| ICD9_527 |  | 0.07 |  | 4 | <0.01 |  | 38 | 0.05 |  | 12 | | 0.01 |  | 2 | <0.01 | 0.4546 |
| ICD9_528 |  | 2.71 |  | 306 | 0.37 |  | 1384 | 1.67 |  | 493 | | 0.59 |  | 68 | 0.08 | 0.2652 |
| ICD9_529 |  | 0.03 |  | 1 | <0.01 |  | 14 | 0.02 |  | 12 | | 0.01 |  | 2 | <0.01 | 0.0105 |
